# Construction and analysis of lncRNA-lncRNA synergistic networks to reveal clinically relevant lncRNAs in cancer

**DOI:** 10.18632/oncotarget.4660

**Published:** 2015-07-28

**Authors:** Yongsheng Li, Juan Chen, Jinwen Zhang, Zishan Wang, Tingting Shao, Chunjie Jiang, Juan Xu, Xia Li

**Affiliations:** ^1^ College of Bioinformatics Science and Technology, Harbin Medical University, Harbin, China

**Keywords:** long non-coding RNA, network hub, functional synergistic network, hallmark of cancer, prognostic module

## Abstract

Long non-coding RNAs (lncRNAs) play key roles in diverse biological processes. Moreover, the development and progression of cancer often involves the combined actions of several lncRNAs. Here we propose a multi-step method for constructing lncRNA-lncRNA functional synergistic networks (LFSNs) through co-regulation of functional modules having three features: common coexpressed genes of lncRNA pairs, enrichment in the same functional category and close proximity within protein interaction networks. Applied to three cancers, we constructed cancer-specific LFSNs and found that they exhibit a scale free and modular architecture. In addition, cancer-associated lncRNAs tend to be hubs and are enriched within modules. Although there is little synergistic pairing of lncRNAs across cancers, lncRNA pairs involved in the same cancer hallmarks by regulating same or different biological processes. Finally, we identify prognostic biomarkers within cancer lncRNA expression datasets using modules derived from LFSNs. In summary, this proof-of-principle study indicates synergistic lncRNA pairs can be identified through integrative analysis of genome-wide expression data sets and functional information.

## INTRODUCTION

Recent analyses of the mammalian transcriptome revealed that an abundance of long non-coding RNAs (lncRNAs) lie interspersed with the coding genes [1]. While the functions of most lncRNAs remain unknown, growing evidence suggests that the like microRNAs (miRNAs), lncRNAs, mediate oncogenic or tumor-suppressing effects and may constitute a new class of cancer therapeutic targets [2, 3]. Moreover, it appears likely that many biological molecules, including miRNAs and lncRNAs, exert their effects by acting in combination rather than individually [4, 5]. But despite the growing appreciation of the importance of lncRNAs in normal physiology and disease, our understanding of the combined effects of the cancer-associated lncRNAs remains limited.

Several recent studies suggest that lncRNAs play important roles in oncogenesis [6, 7]. H19, for example, is an lncRNA induced during liver development [8], but it also promotes glioma cell invasion by giving rise to miR-675 [9]. Other lncRNAs involved in various types of cancers include HOTAIR [10, 11], MEG3 [12], PVT1 [13] and CDKN2B-AS1 [14]. Among those, levels of the lncRNA MEG3 are markedly lower in glioma tissues than adjacent normal tissues, and ectopic expression of MEG3 inhibits cell proliferation and promotes apoptosis [12]. In addition, several earlier findings implicate PVT1 in the pathophysiology of cancer [15], and silencing PVT1 expression using siRNA reduces cell proliferation and increases apoptosis in breast and ovarian cancer cell lines [16]. Another well-known potential cancer-associated lncRNA is PCA3, a prostate-specific molecule that is markedly overexpressed in prostate cancer. A noninvasive assay for urinary PCA3 expression is currently being developed as a clinical diagnostic [17].

Studies focusing the mode of action of lncRNAs suggest an intriguing hypothesis, that lncRNAs serve as flexible modular scaffolds [18–20]. In this model, lncRNAs contain discrete domains that interact with specific proteins. In this way, lncRNAs bring specific regulatory proteins into close proximity to produce a functional complex. In addition, Ma et al. demonstrated that genes are regulated by lncRNAs in part through chromosome conformation capture, which suggests lncRNAs may act synergistically [21]. Although the modular RNA regulatory code remains to be tested, studying the modular regulation of lncRNAs and investigating their combined effects are important steps toward further definition of lncRNA functions on a system-wide level. One approach to classifying the putative functions of lncRNAs is through “guilt-by-association” [22]. This approach associates lncRNAs with biological processes based on a common expression pattern across cell types and tissues, and can therefore identify groups of lncRNAs that are associated with specific cellular processes [23, 24]. Analysis of coexpressed genes of lncRNAs has revealed significant coregulation of genes by lncRNAs, suggesting that lncRNA clusters may regulate biological processes synergistically. It has also been reported that combined lncRNA signatures enable more accurate prediction of patient survival than individual lincRNAs [25, 26]. Results from all of these studies attest to the importance of lncRNA synergism and indicate that integrating expression of correlated genes and functional information could enable identification of synergistic lncRNA pairs and simultaneously reveal their underlying functions.

**Figure 1:**
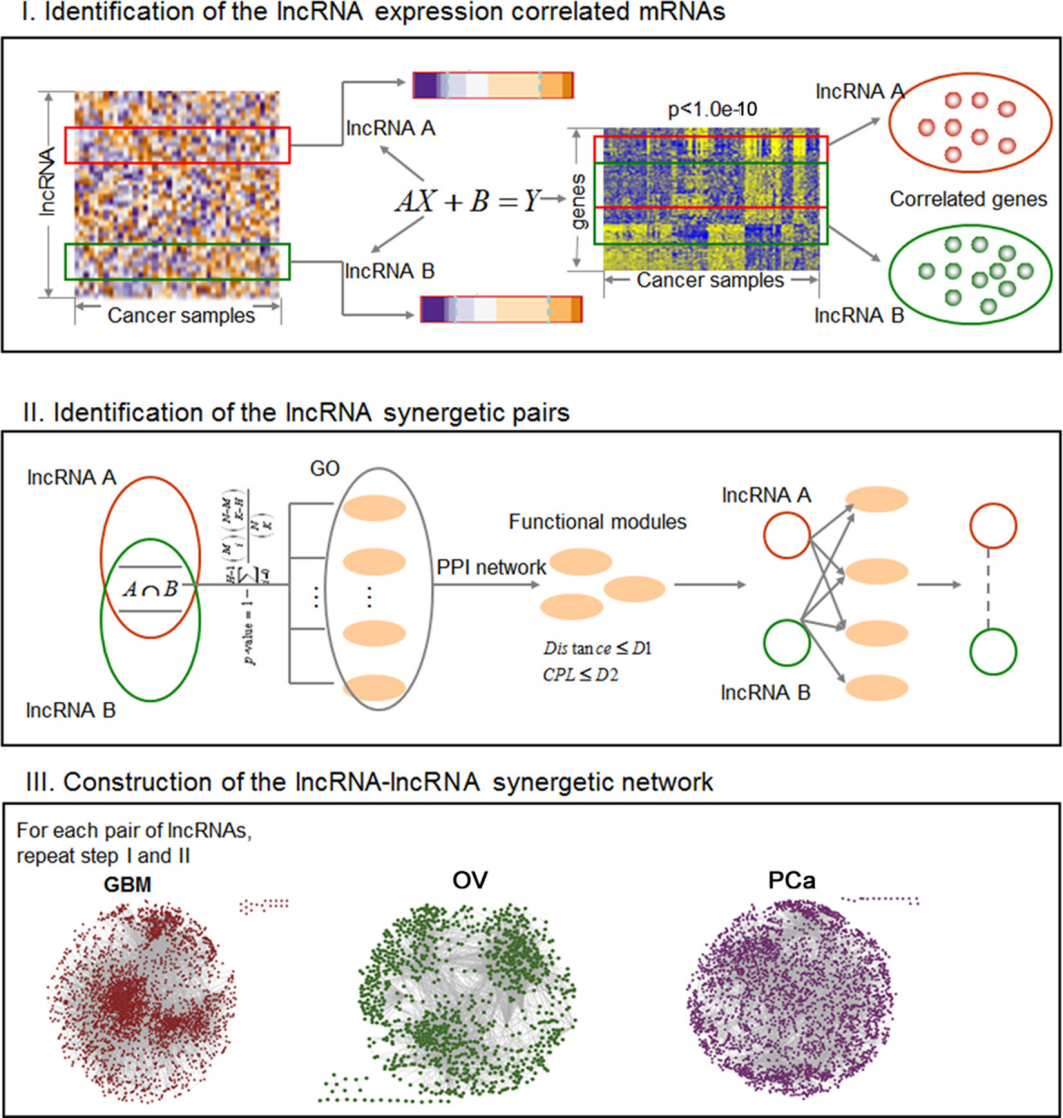
The workflow to construct the LFSNs via co-regulating functional modules The process involves three main steps. First, we identified the coexpressed genes for each lncRNA based on a linear regression model. Second, an lncRNA pair that synergistically regulates at least one functional module was identified. Third, we repeated the first and second steps for any lncRNA pairs, and assembled all the significant lncRNA pairs to construct a LFSN.

Here we present a multi-step computational method for identifying significantly functional synergistic lncRNA pairs through the functional modules they jointly regulate. This entails integrating genome-wide lncRNA and mRNA expression profiles, functional information and protein interaction data. After assembling all the lncRNA-lncRNA functional synergistic pairs, we constructed a lncRNA functional synergistic network (LFSN, our strategy is illustrated in Figure 1). Applying this approach to three cancer types, glioblastoma multiforme (GBM), ovarian cancer (OV) and prostate cancer (PCa), we constructed cancer associated LFSNs having scale free and modular architectures, and investigated the properties of cancer-related lncRNAs within their corresponding synergistic networks. Our findings suggest that cancer-related lncRNAs are enriched in hubs and modules, and that the cancer-related lncRNA modules are associated with cancer prognosis. This study contributes to a comprehensive understanding of the synergistic behaviors of lncRNAs from the viewpoint of systems biology.

## RESULTS

### The lncRNA functional synergistic network (LFSN) across cancers

**Figure 2:**
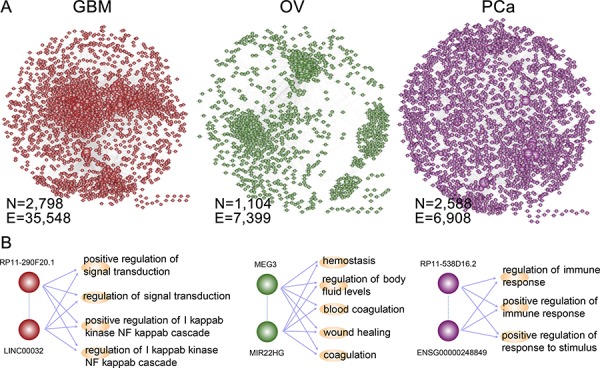
The lncRNA-lncRNA synergistic network across cancers **A.** The LFSNs in GBM, OV and PCa. A node represents a lncRNA, and an edge represents a synergistic action. **B.** Three examples of lncRNA synergistic pairs in each cancer. The known disease lncRNAs with high connectivity were selected as examples. The GO processes regulated by lncRNA pairs were also shown. The indirect dashed line represents the lncRNA synergistic action; the direct line represents the lncRNA regulation of the functional module.

We are proposing a multi-stage method to gradually identify synergistic lncRNA pairs in cancer (Figure 1, see details in methods). On the basis of lncRNA pairs regulating at least one functional module, we assembled all synergistic lncRNA pairs into a LFSN, where nodes represent lncRNAs and edges represent their functional synergistic interactions. We applied this approach to three cancer types, GBM, OV and PCa, and constructed three cancer-specific LFSNs. In total, we obtained 35,584 synergistic interactions among 2,798 lncRNAs for GBM, 7,399 synergistic interactions among 1,104 lncRNAs for OV and 6, 908 synergistic interactions among 2,588 lncRNAs for PCa (Figure 2A and Supplementary Table S1). This indicates that lncRNA pairs identified by our method simultaneously regulate multiple cancer-associated biological processes (BPs), which may contribute to carcinogenesis. In particular, a number of known disease-related lncRNAs with high connectivity within the network were observed to co-regulate the cancer associated BPs. For example, an analysis of genome-wide DNA copy number alterations revealed the loss of LINC00032 in approximately one-fourth of tumors [27]. We observed that LINC00032 co-regulated 285 functional modules with other lncRNAs, including the highly connected lncRNA RP11–290F20.1 in GBM (left panel, Figure 2B). In addition, MEG3 has been shown to activate p53 and inhibit tumorigenesis and disease progression of several types of cancers [28, 29], including OV [30]. We found that MEG3 co-regulated 142 functional modules with other lncRNAs, among which MIR22HG is associated with stress responses [31, 32]. These two lncRNAs act synergistically to regulate coagulation-related functions (middle panel, Figure 2B), which are associated with tissue invasion and metastasis. In PCa, we observed two lncRNAs with high connectivity that co-regulate the immune response (right pane, Figure 2B). The lncRNA RP11–538D16.2 regulated 102 functional modules, while ENSG00000248849 regulated 87 modules. In addition, ENSG00000248849 is subject to recurrent deletion in PCa, and shows significant overlap with a known disease lncRNA DLX6-AS1 [33, 34]. All of these observations demonstrate the feasibility of using our method to identify lncRNA pairs through their synergistically regulating functional modules. Furthermore, the examples summarized above suggest that some disease-associated lncRNAs have especially high connectivity within the LFSN. Understanding the structure of LFSNs may provide additional insight into the tumorigenesis of certain types of cancer.

### Properties of LFSNs

**Figure 3:**
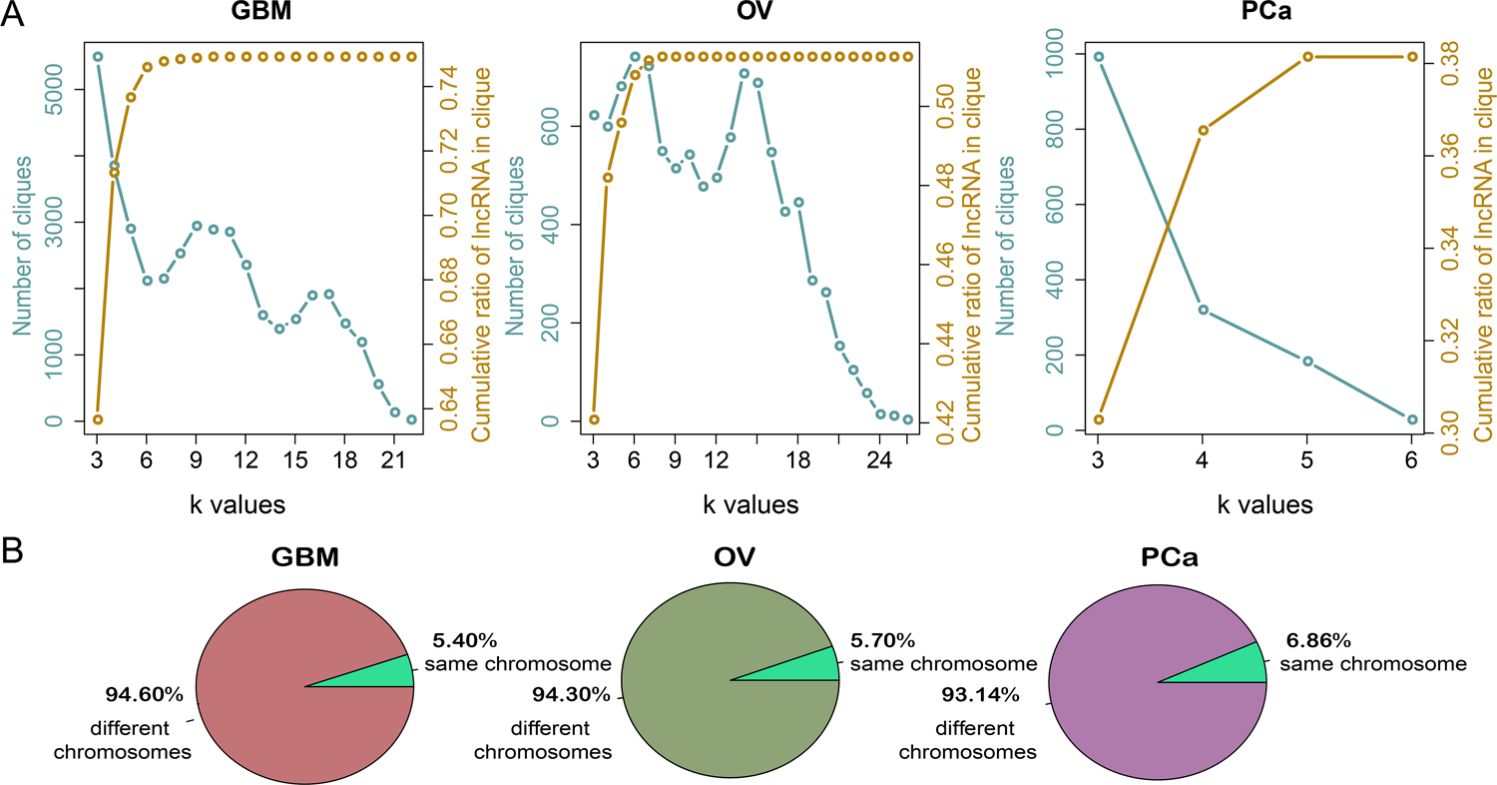
The topological features of the LFSNs across cancers **A.** Number of cliques at different k-values and cumulative ratios of lncRNAs in cliques with k-values are not bigger than k. The left y-axis represents the number of cliques under different k-values, corresponding to the blue line. The right y-axis represents the cumulative ratios of lncRNAs within cliques, corresponding to the yellow line. **B.** The chromosome distribution of lncRNA pairs in cancers.

We initially explored the structure and organization of LFSNs in GBM, OV and PCa. We found that most lncRNAs interact with few lncRNA partners, while a quite few lncRNAs have large numbers of synergistic partners (Supplementary Figure S1A). Investigation of the degree distribution of the cancer-associated LFSNs revealed power law distributions, indicating that the LFSNs are scale free. These observations suggested that like many biological networks, the structures of LFSNs were not randomly organized; instead, they were characterized by a core set of principles that distinguish them from random networks. We next analyzed the modular structure of each LFSN. As shown in Figure 3A, the number of modules decreased with increases in the k-value. We hypothesized that this reflected the tendency of lncRNAs to implement synergistic regulation as small modules rather than as individuals or as parts of larger modules. The same features were observed in OV and PCa. In total, approximately 74.92%, 51.25% and 38.14% of lncRNAs were involved in at least one module in GBM, OV and PCa, respectively.

Exploration of the chromosomes on which the synergistic lncRNA pairs were located showed that only 1,918 (5.39%) lncRNA pairs in GBM, 422 (5.70%) pairs in OV and 474 (6.86%) pairs in PCa were located on the same chromosomes (Figure 3B). Although the number of lncRNA pairs co-localizing on the same chromosome in OV and PCa was small, the relation was significant (*p* = 0.057 and *p* < 0.001, respectively). In addition, 12.62%, 14.22% and 10.13% of pairs locate within less than 10 Mb on the same chromosomes, but these proportions are not significantly higher than random. These results indicate that the majority of the synergistic pairs regulate the same function modules *in trans*, while a small fraction of lncRNA pairs synergistically regulate *in cis*.

### Cancer associated lncRNAs tend to be hubs and enriched in modules

**Figure 4:**
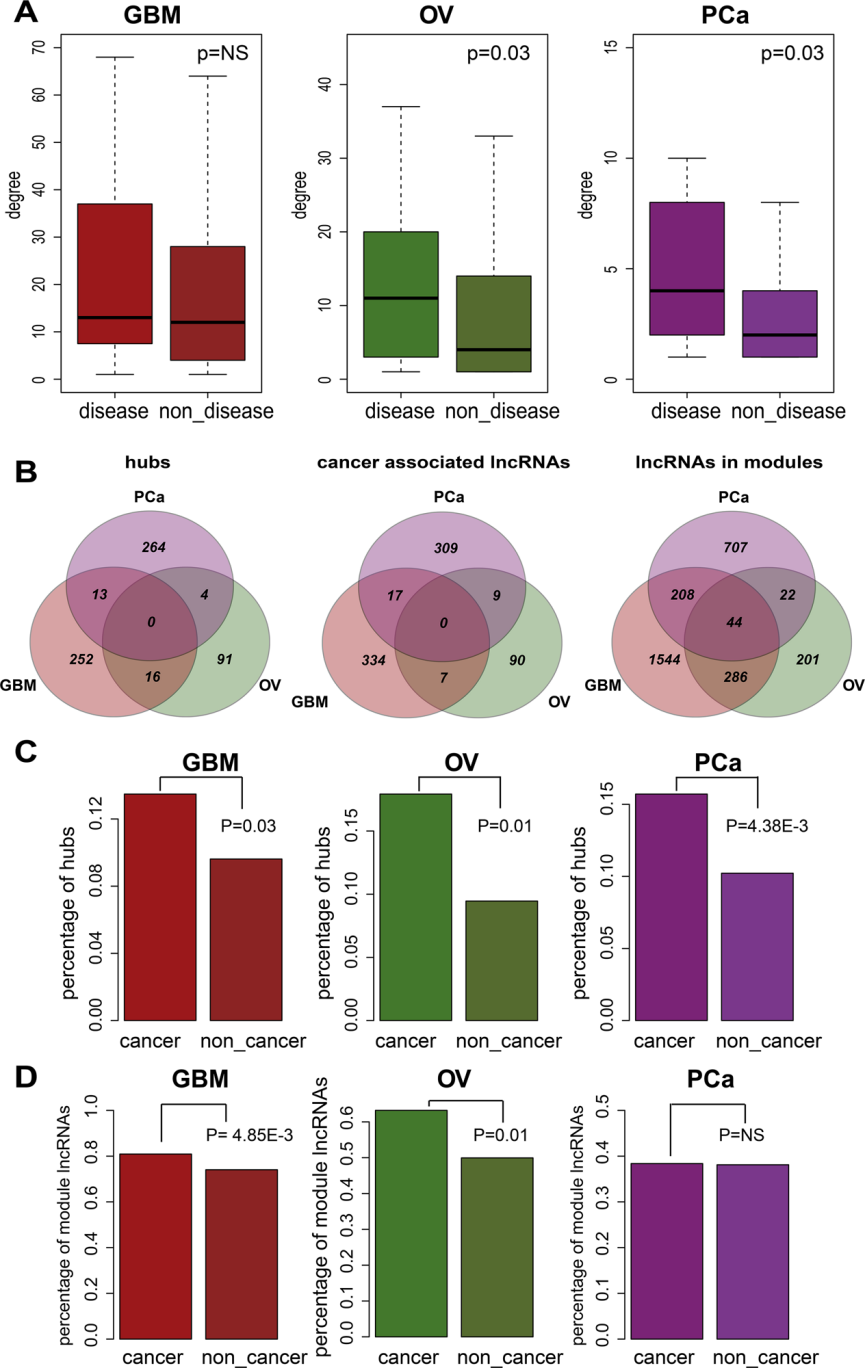
The cancer-associated lncRNAs tend to be hubs and are enriched in modules **A.** The difference in degrees between disease-associated lncRNAs and lncRNAs not related to disease. Light colored boxes represent the distribution of disease-associated lncRNA degrees, and the dark colored boxes correspond to lncRNAs not related to disease. *P*-values were calculated using the Wilcoxon rank-sum test. **B.** Overlap of hubs, cancer-associated lncRNAs and module lncRNAs across three cancers. **C.** Cancer-associated lncRNA are enriched in the hubs. Light and dark colored bars respectively depict the proportions of hubs among cancer-associated and unrelated lncRNAs. *P*-values were calculated using Fisher's exact test. **D.** Cancer-associated lncRNAs are enriched within the modules. Light and dark colored bars respectively depict the proportions of module lncRNAs among cancer-associated and unrelated lncRNAs. *P*-values were calculated based on Fisher's exact test.

For each biological network, a key property is its connectivity, which reflects how often a node interacts with other nodes. Hub nodes whose connectivities are extremely high are always very important nodes. For example, in the protein-protein interaction networks of various organisms, hubs tend to be essential proteins involved in maximal information exchange with other proteins [35]. We focused on lncRNAs that had been experimentally validated as disease lncRNAs. Notably, the connectivities of these disease lncRNAs were significantly greater than the connectivities of other lncRNAs within the entire network (Figure 4A, Wilcoxon rank sum test). This suggests disease lncRNAs are highly connected within the network and may be enriched in hubs. Because the number of experimentally validated disease lncRNAs is limited, we extended this set by identifying cancer-associated lncRNAs in each cancer type (details in Methods). Generally, the hub and cancer-associated lncRNAs defined in the three cancer networks examined were almost entirely separate with little overlap (Figure 4B). But When we tested whether the cancer-associated lncRNAs were more likely to be hubs, which we defined as the top 10% of nodes with the highest connectivity within each network, we found that cancer-associated lncRNAs were significantly enriched in the hubs across all three cancers tested (Figure 4C, Fisher's exact test).

Another important feature of a biological network is modularity. Modules are groups of highly interconnected nodes that are often involved in the same biological processes or pathways. In contrast to hubs and cancer-associated lncRNAs, we found substantial overlap of module lncRNAs across the three cancer types (Figure 4B). This is reasonable and understandable since modules mainly reflect their underlying biological processes. The cancer-associated lncRNAs showed a significant enrichment in modules across GBM and OV (Figure 4D, Fisher's exact test). This suggests that cancer-associated lncRNAs work more synergistically and reflect the group behavior, making it likely that they are more informative regarding tumor initiation and progression.

### Synergistic lncRNAs modulate cancer-associated hallmarks

Although the biology of cancer is extremely complex, that complexity can be reduced and represented by a few cancer hallmarks that enable tumor growth and metastasis [36]. These hallmarks provide a framework for understanding the remarkable diversity of cancers. To further understand the functional roles of synergistic lncRNA pairs in carcinogenesis, for each LFSN, cancer hallmarks associated lncRNA pairs were selected to form cancer hallmark subnetworks. This analysis showed that several cancer hallmarks were deeply involved in the three LFSNs (Supplementary Figure S2), but with different cancer specificities. In brief, lncRNA pairs in GBM primarily regulate the cancer hallmarks of “self sufficiency in growth”. Previous studies have demonstrated that GBM is characterized by extensive invasion, rapid growth, necrosis and angiogenesis [37, 38]. The lncRNA pairs in OV mostly regulate the hallmarks “self sufficiency in growth” and “tissue invasion and metastasis”, which is consistent with clinically rapid progress and high metastatic potential of ovary tumors [39, 40]. In addition, “evading immune detection” and “tumor promoting inflammation” are the two most affected hallmarks in PCa, which indicates that inflammation and immune processes are important for the malignancy of prostate tissue. This is consistent with histological observations, indicating that innate and adaptive immunity actively participates in the pathogenesis, surveillance and progression of prostate cancer [41–43].

Although no synergistic lncRNA pair was found to share by any two hallmark subnetworks, the hallmark subnetworks are conserved among the three cancers to a certain extent. That is, a large percentage of lncRNA pairs contribute to the same cancer hallmarks by regulating biological processes annotated to those hallmarks. For example, 29, 11 and 689 lncRNA pairs in GBM, OV and PCa, respectively, regulate the biological process of “DNA repair”, which is related to the “genome instability and mutation” hallmark (Supplementary Figure S3). For GBM and OV, 108 and 97 pairs of lncRNAs regulate “positive regulation of cell proliferation” (Supplementary Figure S4). In PCa, there are no lncRNA pairs regulating this biological process, but three lncRNA pairs control another process, “positive regulation of signal transduction” and both processes are annotated to the cancer hallmark “self sufficiency in growth”. Within these cancer hallmark subnetworks, some experimentally validated disease lncRNAs contribute to carcinogenesis. In GBM, TRAF3IP2-AS1, which is related to brain-associated disease, cooperates with three lncRNAs to regulate “wound healing”: AC078883.3, RP11-645N11.2 and XLOC_004923. In OV, the obesity-associated lncRNA SNHG11 cooperates with three lncRNAs, RP11-79H23.3, XLOC_002749 and XLOC_0092721, to regulate “positive regulation of cell proliferation” which is annotated to the hallmark of “self sufficiency in growth”. In PCa, the lncRNA MIAT functions together with AC005562.1 to regulate “DNA repair” and so impacts the hallmark of “genome instability and mutation”. In addition, for a specific cancer, the same lncRNA synergistic pairs may regulate different processes to control cancer growth. In GBM, for example, ENSG00000248849, which shows 80% overlap with the disorder-associated lncRNA DLX6-AS1, cooperates with RP5-1185K9.1 to regulate the processes “negative regulation of apoptosis” and “negative regulation of programmed cell death”, both of which annotated to the cancer hallmark “evading apoptosis”. These results indicate that lncRNA pairs in each cancer hallmark subnetwork may contribute to carcinogenesis and deserve further investigation across these three cancer types.

### Clinically relevant lncRNA synergistic modules

The notion that it may be possible to reduce cancer mortality by identifying and monitoring survival-related biomarkers rests on the idea that a module biomarker is a better predictor of survival than an individual gene [44]. Given than lncRNA pairs within hallmark subnetworks were associated with cancer-related biological processes, we were interested in identifying prognostic modules from the cancer hallmark subnetworks. We therefore used the Markov Clustering Algorithm (MCL) method with suggested parameters to identify modules in each hallmark subnetwork. As a result, 43, 13 and 15 modules were identified in GBM, OV and PCa, respectively. Among these lncRNA modules, 12 and 2 in GBM and OV can be used to classify cancer samples into two groups with significantly different overall survival rates (log-rank test, *P* < 0.05). Furthermore, the gene or miRNA expression patterns can be used to segregate those two diseases into subtypes with different prognoses [45, 46]. However, it is not yet clear whether the classifier power of these modules reflects their correlation with global expression subtypes in GBM or OV. To determine whether these functional modules can provide additional predictive power, we performed a multivariate survival analysis using prognostic factors, such as age, sex, subtype, grade or stage of patients, along with subnetwork modules. We observed that four lncRNA modules in GBM and two modules in OV were significantly associated with patient survival (Supplementary Table S2–S4).

**Figure 5:**
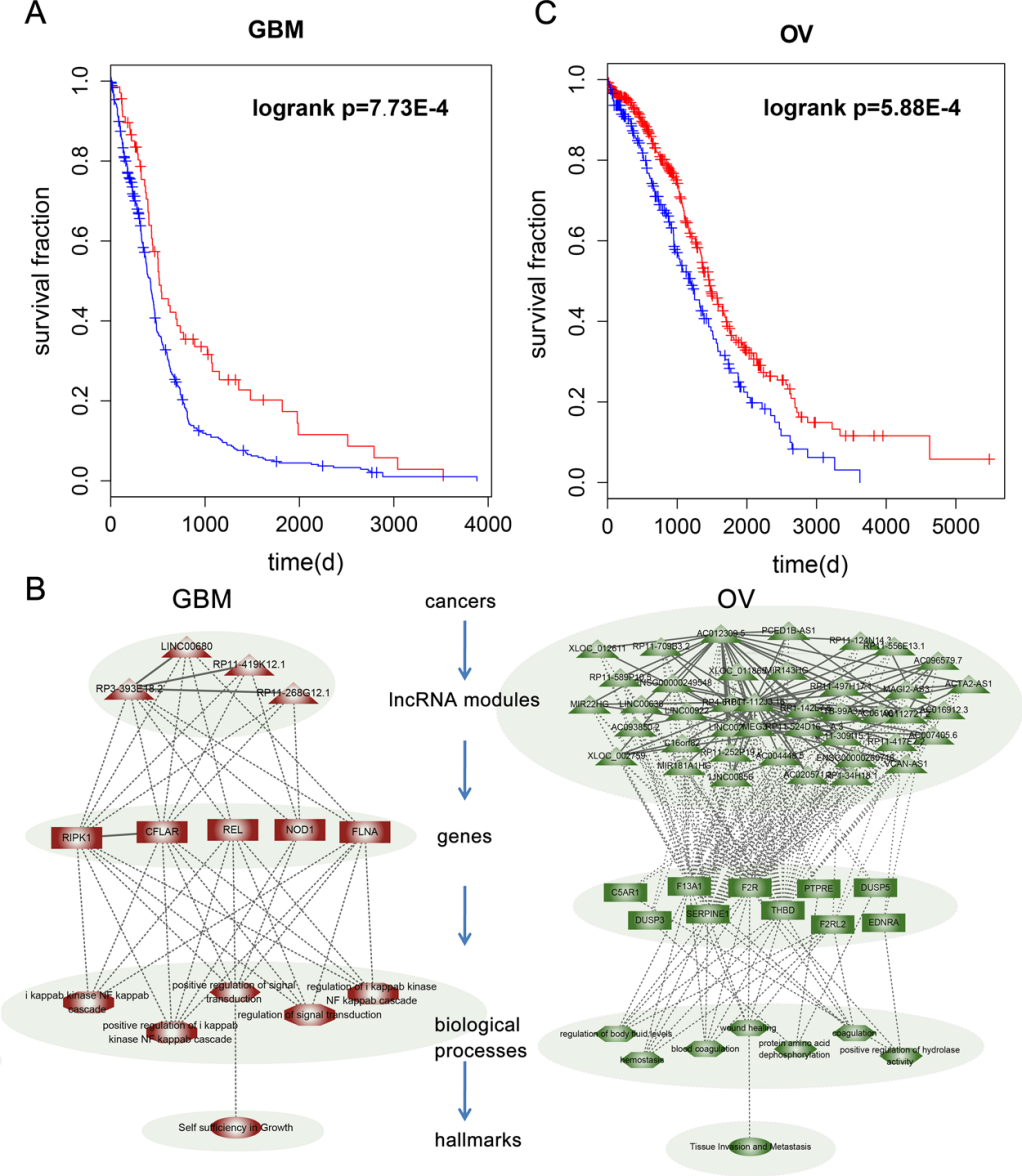
The prognostic lncRNA modules in cancer **A.** Kaplan-Meier survival curves for GBM patients classified into high- and low-risk groups using the lncRNA module expression signature. *P*-Values were calculated using the log-rank test. **B.** LncRNA modules in GBM and OV. The coexpressed genes, GO terms enriched by the genes and cancer hallmarks are also shown in the hierarchical model. **C.** Kaplan-Meier survival curves for OV patients classified into high- and low-risk groups using the lncRNA module expression signature.

A representative prognostic module in GBM found to be associated with patient survival time (*P* = 7.73E-4, Figure 5A), included three synergistic pairs among four lncRNAs (Figure 5B). This module remained significantly associated with patient overall survival (Hazard Ratio = 0.65, *p* = 0.021) in a Cox multivariate analysis, after adjusting for patient age, gender and tumor subtype. The module includes the lncRNA RP11-419K12.1, which overlaps an experimentally validated GBM-associated lncRNA, CCDC26. A single nucleotide polymorphism (SNP) rs891835 in CCDC26 is reportedly associated with glioblastoma susceptibility. Analysis of the genes co-regulated by these four lncRNAs showed that they are mainly involved in signal transduction and included RIPK1, CFLAR, NOD1 and FLNA. This suggests identification and characterization of signal transduction pathways altered by lncRNA synergistic pairs may contribute to identification of therapeutic targets and enable more effective treatment in GBM. A module consisting of 91 synergistic pairs among 41 lncRNAs was associated with survival in OV (*P* = 5.88E-4, Figure 5C). Cox multivariate analysis indicated that this module was significantly associated with overall patient survival (Hazard Ratio = 0.7, *p* = 0.004) after adjusting for patient age, grade and tumor stage. Among these lncRNAs, MEG3 is affected in many cancer types, including cervical and prostate cancer. Another lncRNA in the module ENSG00000249548 shows more than 80% overlap with an experimentally validated PCa associated lncRNA, CTBP1-AS. Moreover, some lncRNAs in this module, such as MIR22HG [47] and MIR181A1HG [48], were found to be host genes for miRNAs known to play critical roles in various types of cancer.

Evidences also shows that activation of PAR1 (F2R) induces changes in cell morphology associated with increased cell motility and plays a critical role in cancer cell invasion and metastasis [49]. In addition, DUSP3 has been implicated in human cancer, though it has been alternatively described as having tumor suppressive and oncogenic properties [50]. Functional analysis of these genes revealed that this module mainly regulates coagulation-related functions (Figure 5B). In the context of cancer biology, hemostatic components are interconnected in multiple ways. Consequently, while cancer cells are able to activate the coagulation system, hemostatic factors also play a role in tumor progression. This analysis potentially opens the way to development of bifunctional therapeutic approaches that capable of both attacking malignant processes and resolving coagulation impairment.

Collectively, our examples suggest that analysis of the structure of LFSNs is a simple and efficient method for detecting prognostic biomarkers in cancer lncRNA expression datasets using modules derived from the LFSNs.

## DISCUSSION

In this study, we presented a multi-step method for constructing the LFSNs based on co-regulating functional modules, which enable an in-depth analysis of lncRNA regulation on a system-wide level. We applied this approach to three types of cancer and performed a systematic analysis of the properties of cancer-associated lncRNAs in the context of LFSNs. Although the LFSNs, cancer-associated lncRNAs and hub membership within the networks varied greatly from one cancer to another, our study revealed several distinct properties of cancer-associated lncRNAs, and the consistency of observed patterns across multiple cancers highlights their robustness. As general biological networks, LFSNs are scale free and modular. Hub lncRNAs are topologically central within LFSNs and have maximal informational connections with other lncRNAs. In addition to their high overall connectivity, we found cancer-associated lncRNAs to be enriched in the hub nodes of cancer-specific LFSNs. As hubs are associated with very high levels of activity involving both the receiving and sending of signals, disruption of their expression may contribute to the progression of cancers.

Cancer-associated lncRNAs are enriched within modules, which are groups of functionally related lncRNAs dedicated to specific biological processes. Compared to single prognostic lncRNAs, biological lncRNA modules may provide more robust prognostic signatures. Based on the topological structures of LFSNs, we described a simple and rapid procedure for combining disease-specific lncRNA expression data in order to identify candidate prognostic network modules. From a clinical viewpoint, it would be much more applicable if one biomarker could be detected in serum or other body fluid. With that in mind, we reannotated the probes in the microarray provided by Noerholm et al. [51] for these candidate lncRNAs and observed that 54.55% of lncRNAs within the modules were detected in serum. In particular, four lncRNAs within the modules were differentially expressed in GBM (*P* < 0.1, Student's *t*-test). In addition, using the RNA-Seq dataset for OV from one recent study [52], we found that 92.68% of lncRNAs within the modules (Figure 5B) were detected and 63.16% were differentially expressed in OV (*P* < 0.05, Student's *t*-test). These results suggest that the majority of lncRNAs are detectable in serum or ascites and may be applicable for clinical trial.

In our present study, we used a common simplification process to facilitate analysis of the topological structure of the LFSNs and to identify central lncRNAs and lncRNA modules within the LFSNs. This entailed setting the weights of all edges to 1. In addition, we also tried to consider the co-regulating strength for each lncRNA pair based on the number of shared modules. Analysis of the structure of the weighted LFSNs yielded results simiar to those obtained earlier and the degree distributions of the networks also followed power-law distributions (Supplementary Figure S1B). Similarly, the top 10% lncRNAs with the highest summary scores were defined as central lncRNAs within the weighted LFSNs, and the modules were further identified under different weight thresholds. Cfinder was also used to detect modules, and four different weight gradients were considered in GBM and OV. Because most synergistic lncRNA pairs in PCa shared no more than two functional modules, we analyzed only two weight gradients for PCa. The weighted networks continued to show modular structures (Supplementary Figure S5), and cancer-related lncRNAs were enriched in the central lncRNA sets (Supplementary Figure S6 and S7). In summary, these results are indicative of the robustness of the structures of LFSNs and the topological features of cancer-associated lncRNAs.

We not only identified the synergism among lncRNAs, we also revealed their underlying functional patterns. LncRNAs are a new class of ncRNAs functioning as regulators of gene expression, and their dysregulation of lncRNAs and the resultant dysregulated gene expression, has been shown to play critical roles in cancer. We have shown here that lncRNAs carry out their functions by acting in combination and proposed a hierarchical model to systematically understand the lncRNA regulatory networks in human cancers (Figure 5B). Four kinds of nodes were included in the model: lncRNA modules, genes, GO terms and cancer hallmarks. All of the illustrated examples suggest that lncRNA pairs function synergistically through regulation of co-correlated genes to further impact cancer-related singaling pathways, thereby mediating tumor initiation and development.

Although our study provides a degree of insight into the emergent properties of cancer-related lncRNAs in the context of LFSNs, additional effort will be required to validate and extend our findings. A univariate linear regression model was used here to identify potential a lncRNA-correlated mRNA subset. The co-expression of lncRNAs and protein coding genes may reflect a variety of scenarios. For example, the genes may co-localize within the genome or may be co-regulated by the same transcription factors, or the lncRNA may be regulated by protein coding genes. To exclude these scenarios, we excluded co-localized and co-regulated genes from the lncRNA-correlated genes (details in Methods). Only 0.16% of lncRNA-gene pairs in GBM were co-localized or co-regulated by transcription factors, and only 0.36% in OV and 0.07% in PCa. After excluding these pairs from the lncRNA-gene pairs, we observed that all of the lncRNA pairs within the LFSNs also shared the common correlated genes. A large majority (99.90% in GBM, 98.34% in OV and 98.82% in PCa) of candidate functional modules co-regulated by lncRNA pairs in the LFSN were the same as in our earlier analyses. In addition, we believe that stringent functional enrichment analysis of GO biological processes and the filtering of candidate functional modules in the PPI network in later steps will compensate for this limitation, increasing our confidence in the functional synergistic lncRNA pairs we identified.

We are only beginning to understand the mechanism by which lncRNAs carry out their regulatory functions. A modular RNA regulatory code is an attractive hypothesis but remains to be tested. Understanding these principles will require identification of lncRNA-protein interactions. At present, however, the lncRNA-protein interaction dataset is limited [53]. Alternatively, we anticipate that by repurposing the publically available lncRNAs and protein-coding gene expression profiles, we will be able to accurately model the combinatorial functions of lncRNAs.

In conclusion, our research provides new insight into the properties of lncRNA regulation through construction of lncRNA functional synergistic networks at the system level. Although limitations exist with the current methods and datasets, the results presented here shed light on the key roles played by synergistic lncRNA regulation within complex diseases.

## MATERIALS AND METHODS

### Datasets

#### lncRNA and mRNA expression profiles across cancers

The genome-wide paired lncRNA and mRNA expression profiles in cancers were obtained from a recent study that repurposed publically available array-based data [54]. Briefly, the exon array data was downloaded from TCGA (https://tcga-data.nci.nih.gov/), after which probe sets for the Human Exon array were reannotated to the human genome (hg19). The expression level of a lncRNA or mRNA was calculated by summarizing the background-corrected intensity of all probes mapped to this gene. The expression of lncRNA and mRNA was quantile normalized across different biological samples, and Combat was used to remove potential batch effects [55]. This procedure yielded the expression of 10, 207 lncRNAs and 18, 292 mRNAs. All the expression profiles were log2 transformed. In our present study, three cancers were analyzed, including 450 samples of GBM tumor tissue, 584 samples of OV and 150 samples of PCa. Twenty-nine samples of healthy tissue were also analyzed. In addition, overall survival data for 419 GBM samples and 558 OV samples were downloaded from Synapse, the TCGA Pan-Cancer website [56] (https://www.synapse.org/).

#### Functional annotation of human genes

The 825 Biological Process (BP) terms for Gene Ontology (GO) were downloaded from the Molecular Signatures Database (MsigDB) [57]. As in previous studies, process categories from GO are restricted to BP terms such that the number of genes annotated to a term is at least 5 and no more than 500. Ultimately, 792 GO BP terms that passed the filtering criteria were taken into account.

#### Human protein-protein interaction network

Protein-protein interaction data was assembled from the HPRD (Human Protein Reference Database) [58] and self-loop interactions were removed. The gene symbols for each interaction were mapped to corresponding Entrez gene identifiers. We extracted the maximum component of the whole protein interaction network, which contained 35,865 interactions among 9,028 genes.

### Methods

#### Overview of the construction of lncRNA-lncRNA functional synergistic network

A three-step model was proposed to construct lncRNA-lncRNA functional synergistic networks in cancer (Figure 1). First, for each lncRNA, a univariate linear regression model was used to identify lncRNA-expression correlated genes (LCGs). Then the functionally synergistic lncRNA pairs were identified as follows: for each lncRNA pair, we initially identified lncRNA pairs that significantly share the LCGs. Second, the shared LCGs of a lncRNA pair were used to identify candidate functional modules, after which the candidate module sets were further filtered using two topological features in the protein interaction network. Here we defined a pair of lncRNAs as synergistic if they significantly co-regulated at least one functional module. Finally, all synergistic lncRNA pairs were assembled into a LFSN, where nodes represented lncRNAs and edges represented their functional synergistic interactions.

#### Identification of expression-correlated lncRNA genes

Although the function of most lncRNAs is unknown, a number of studies have shown that lncRNAs mainly carry out their functions via expression-correlated genes. As in earlier studies, we adopted a univariate linear regression model to identify the expression-correlated genes for specific lncRNAs. For each lncRNA, we assembled all mRNAs that were significantly associated with the expression of the lncRNA of interest at a significance level of 1.0E-10, which approximates the Bonferroni correction FDR = 0.05 (0.05/(10, 207*18,292).

#### Identification of lncRNA-lncRNA synergistic pairs

For a given pair of lincRNAs, *A* and *B*, we identified the common LCGs with which they co-correlated, and a subset of common LCGs was required to have at least three genes. The biological processes enriched by the common LCGs were then identified by hypergeometric distribution. The probability for the common LCGs for a GO term *i* was calculated according to

$$p_{i}=1-F\left(x/N,K_{i},M\right)=1-\sum_{t=0}^{x-1}\frac{\left(\begin{array}{c} K_{i}\\ t \end{array}\right)\left(\begin{array}{c} N-K_{i}\\ M-t \end{array}\right)}{\left(\begin{array}{c} N\\ M \end{array}\right)}\left(i=1,2,\ldots ,I\right)$$

where *N* is the number of all genes, *K_i_* is the number of genes annotated in the GO term *i*, *M* is the size of the common LCGs, *x* is the number of common LCGs also annotated to term *i* and *I* is the total number of GO terms we considered. At a given significance level, we not only obtained the enriched GO terms but also captured the subset of common LCGs annotated to each term, *G_AB_*. Namely, *G_AB_* is the set of candidate function modules. We next further filtered these candidate function modules using two topological features of the protein interaction network: (i) the minimum distance from every gene to others in the subset is no larger than 2; (ii) the characteristic path length (CPL) is significantly shorter than random. The significant *p*-value was calculated using the edge-switching method and was defined as the fraction of the CPL for the same subset in random networks that was shorter than that in the real network. We generated 1,000 random networks using the software Mfinder [59]. After performing function enrichment and two topological restrictions in the network, lncRNAs *A* and *B* were considered to be synergistic if they co-regulated at least one functional module.

#### Construction of the lncRNA-lncRNA functional synergistic network

Finally, a LFSN was constructed for each cancer by assembling all significant lncRNA pairs identified above. A node represents a lncRNA, and two nodes are connected if they are functionally synergistic.

#### Topological measurements of the LFSN

In this study, we investigated several topological features of LFSN using the R package “igraph.” First, we evaluated whether the degree distribution of the synergistic network satisfied a power law model. The degree of connectivity of a node was defined as the total number of edges connecting the node, and the top 10% of the lncRNAs with the highest connectivity within the network were defined as hub nodes. Next, lncRNA functional synergistic modules, which were defined as cliques, were identified in each LFSN using the clique percolation clustering method. Cliques are all of the complete subgraphs, and all cliques in the LFSN were identified using igraph. Each module may overlap, allowing the same lncRNAs or the same interactions to occur in more than one module.

#### Collection of cancer-associated lncRNAs across cancers

We obtained experimentally validated disease lncRNAs from a previous study, including disease lncRNAs in the LncRNADisease database [60], and manually searched from the published literature by our group [61]. All of these known cancer-associated lncRNAs were mapped to the lncRNAs in a microarray based to their genomic positions, and lncRNAs that had at least 80% reciprocal overlap with the known lncRNAs were regarded as experimentally validated cancer lncRNAs. The chromosome locations of disease lncRNAs and 10,207 lncRNAs used here were checked for overlap using BEDTools (−f set to 0.8 here), which yielded 67 experimentally validated disease lncRNAs. There were 32 validated disease lncRNAs in GBM, 21 in OV and 18 in PCa. As the experimentally validated cancer lncRNAs were relatively limited, cancer-associated lncRNA sets were extended in two ways: prognosis-related lncRNAs in GBM and OV or differentially expressed lncRNAs in PCa. The cancer-associated lncRNAs with raw Wald *P*-values < 0.05 generated from the univariate Cox regression model in GBM and OV were added. As clinical information about prostate cancer was not present on Synapse at the time of analysis, we identified the most differentially expressed ones as cancer-associated lncRNAs in PCa (FDR < 0.01, Student's *t*-test with Bonferroni correction). With this approach, we identified 890 cancer-associated lncRNAs in GBM, 683 in OV and 991 in PCa.

#### Hub and module analysis of cancer associated lncRNAs

A Wilcoxon test was used to evaluate differences in the connectivity of disease lncRNAs and other lncRNAs within the whole network. Here we considered cliques as modules. For each cancer type, we used a Fisher's exact test to assess the enrichment of cancer-associated lncRNAs in hub nodes and modules.

#### Cancer-associated hallmarks

A list of GO terms defined to be related to the hallmarks of cancer was obtained from a previous study [62]. Within the LFSNs, we selected the lncRNA pairs whose common correlated genes were significantly enriched in cancer hallmark-related GO terms, then these lncRNA pairs were assembled into a cancer hallmark-related subnetwork. For each cancer hallmark, we calculated whether or not the cancer-associated hallmark was present in each LFSN. We considered it present when there was at least one enriched GO term also related to the cancer hallmark.

#### Identification of clinically related lncRNA modules

We used the Markov Clustering Algorithm (MCL) method to identify modules in each hallmark subnetwork. Then to assess the clinical relevance of these modules in GBM and OV, tumor samples were classified into clusters based on the expression of lncRNAs in modules using K means agglomerative clustering (*K* = 2). The prognostic modules were then identified by testing the correlations of sample clusters with patient survival (log-rank tests, *P* < 0.05).

#### Identification of genes co-localized and co-regulated with lncRNAs

The protein-coding genes within 5 kb of lncRNAs were regarded as being co-localized with the lncRNAs. In addition, we downloaded the TF-gene and TF-lncRNA regulations from the ChIPBase database [63] and then used linear regression to identify the active TF-gene and TF-lncRNA regulations in each cancer (FDR < 0.01, Supplementary Table S5). As in an earlier study [64], two overlap ratios were calculated for a protein coding gene A and a lncRNA B with different numbers of TFs: the proportion of TFs regulating A that were also regulating B (r_AB_), and the proportion of genes regulating B that were also regulating A (r_BA_). We chose the formula r = (r_AB_*r_BA_)^0.5^ to describe the degree of coregulation. LncRNA-gene pairs with an r greater than 0.8 were regarded as co-regulated.

## SUPPLEMENTARY FIGURES AND TABLES






