# Supplementary material for: Construction and analysis of lncRNA-lncRNA synergistic networks to reveal clinically relevant lncRNAs in cancer
Source: Oncotarget. 2015 Jul 28;6(28):25003–16. doi: 10.18632/oncotarget.4660 (PMC4694810; doi:10.18632/oncotarget.4660)
Supplement: Supplementary file 1 [file oncotarget-06-25003-s001.pdf]

SUPPLEMENTARY FIGURES AND TABLES

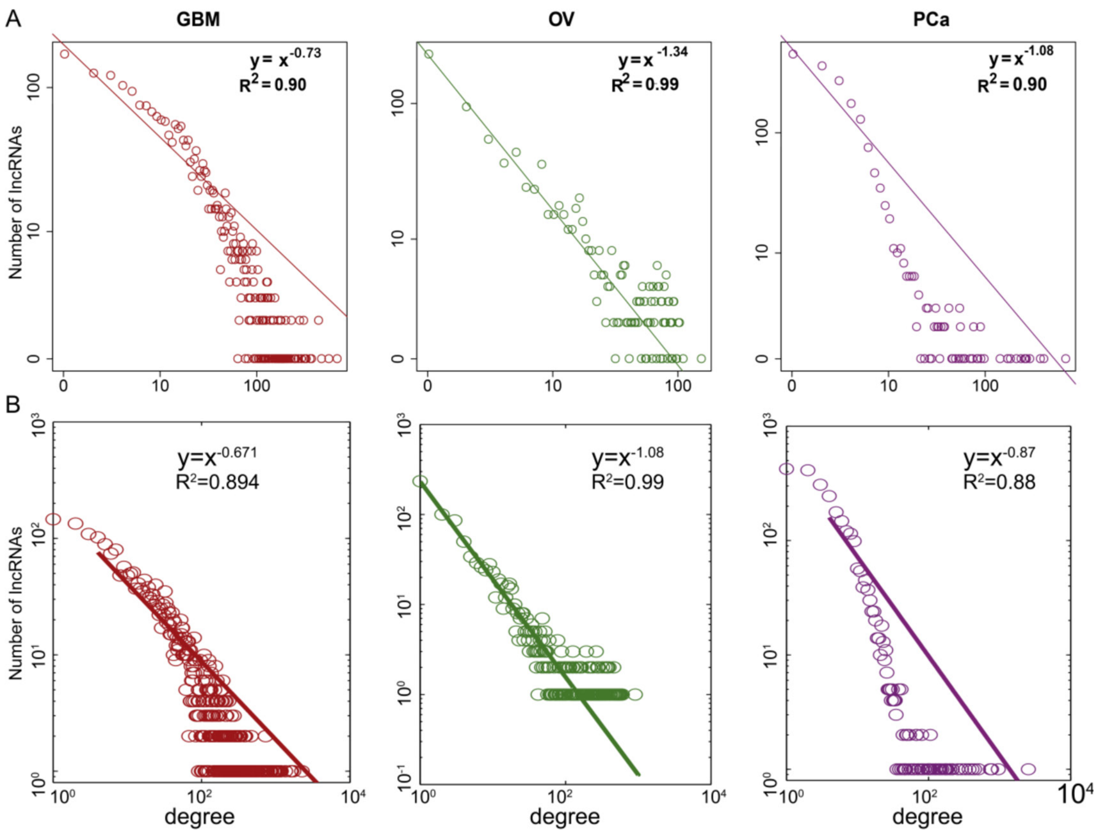

Supplementary Figure S1: The degree distribution of lncRNAs across three types of cancers. A. the degree of lncRNAs were computated based on unweighted networks. **B.** the degree of lncRNAs were computated based on the weighted networks.

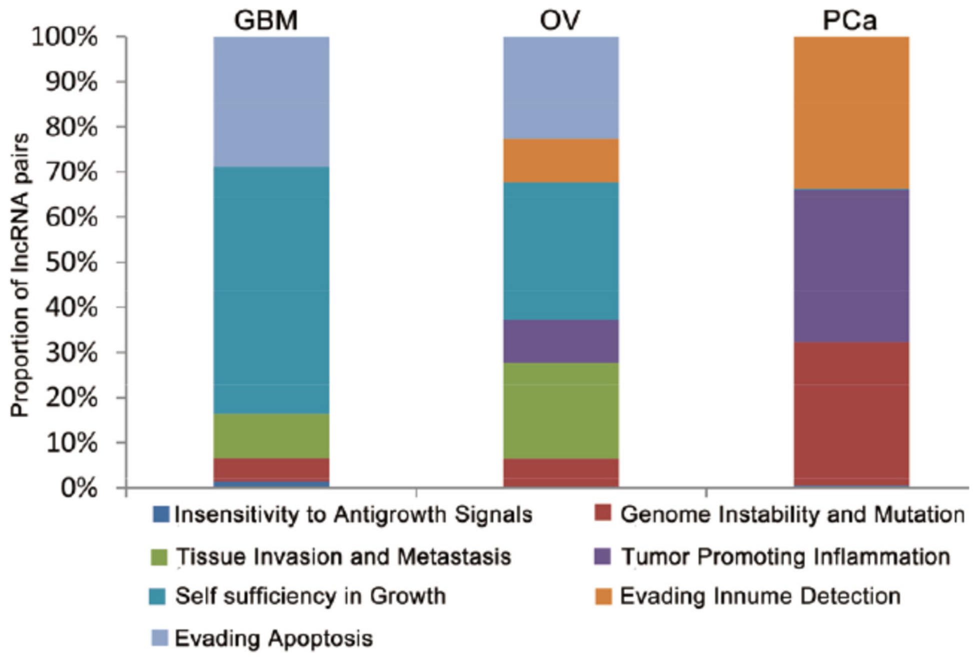

Supplementary Figure S2: The proportion of lncRNA pairs associated with the hallmark of cancers.

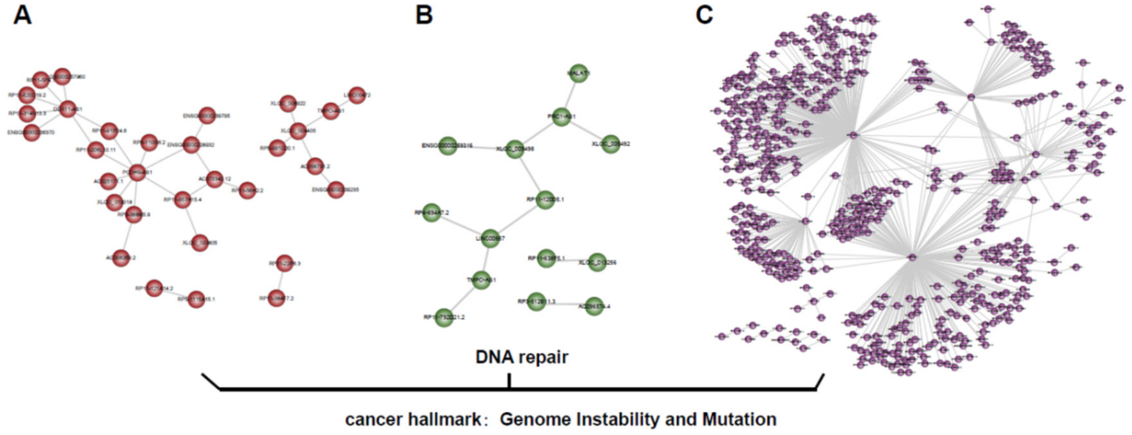

Supplementary Figure S3: The DNA repair subnetwork of lncRNA pairs in cancers. A. GBM, **B.** OV, and **C.** PCa.

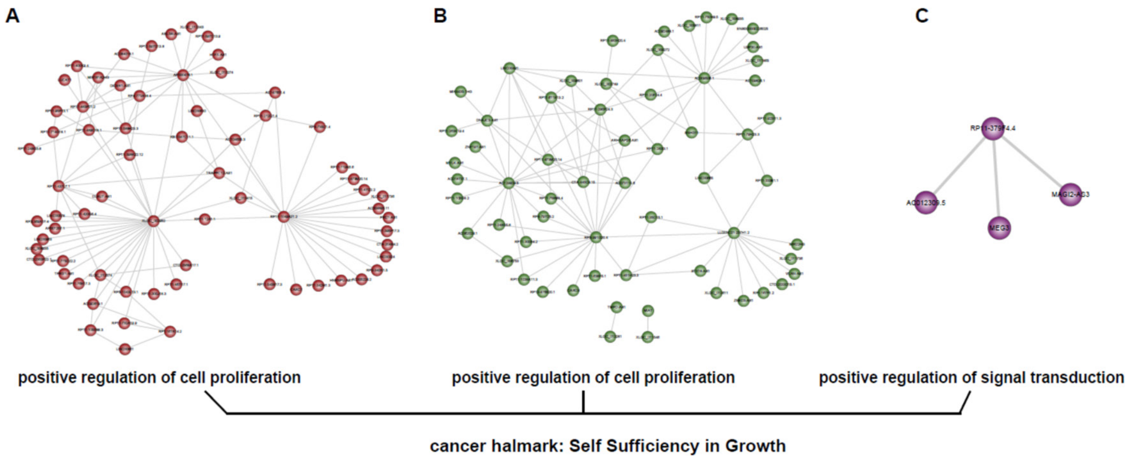

Supplementary Figure S4: The self sufficiency in growth subnetwork of lncRNA pairs in cancers. A. GBM, **B.** OV, and **C.** PCa.

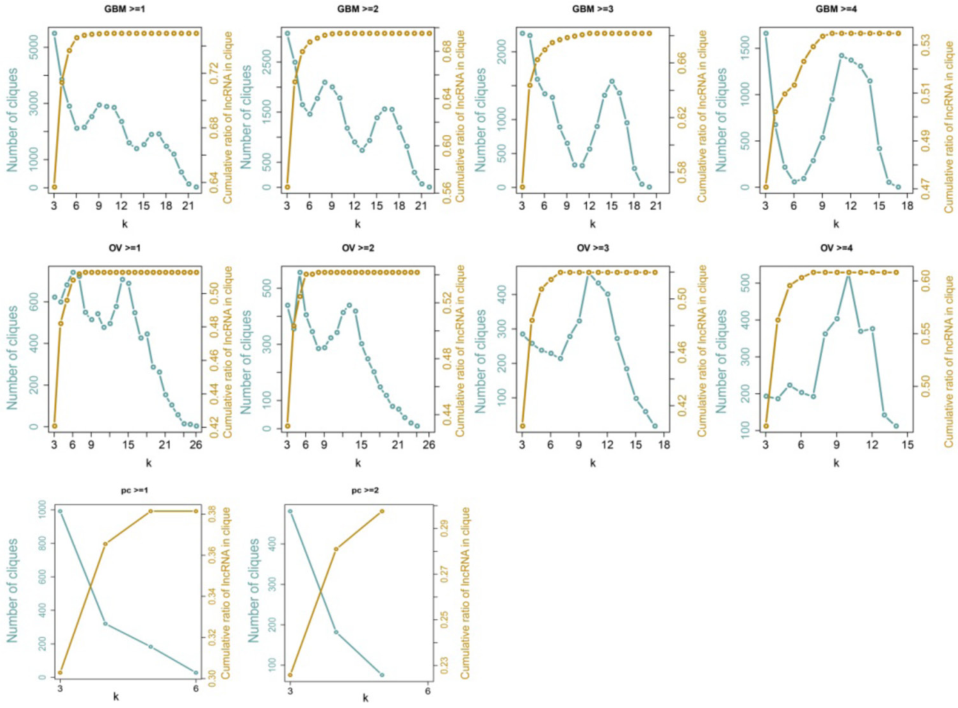

Supplementary Figure S5: The modular structures of the weighted networks in GBM, OV and PCa. The subplots show the modular structures of LFSNs at different threshold of the weights.

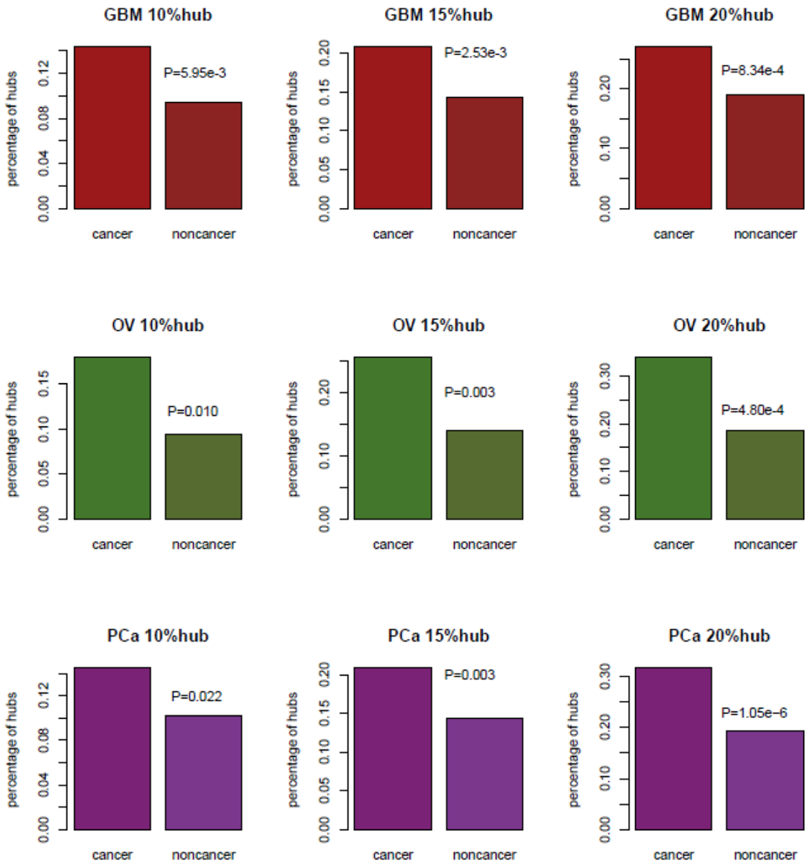

Supplementary Figure S6: The cancer associated lncRNAs are enriched in the hubs in the weighted networks.

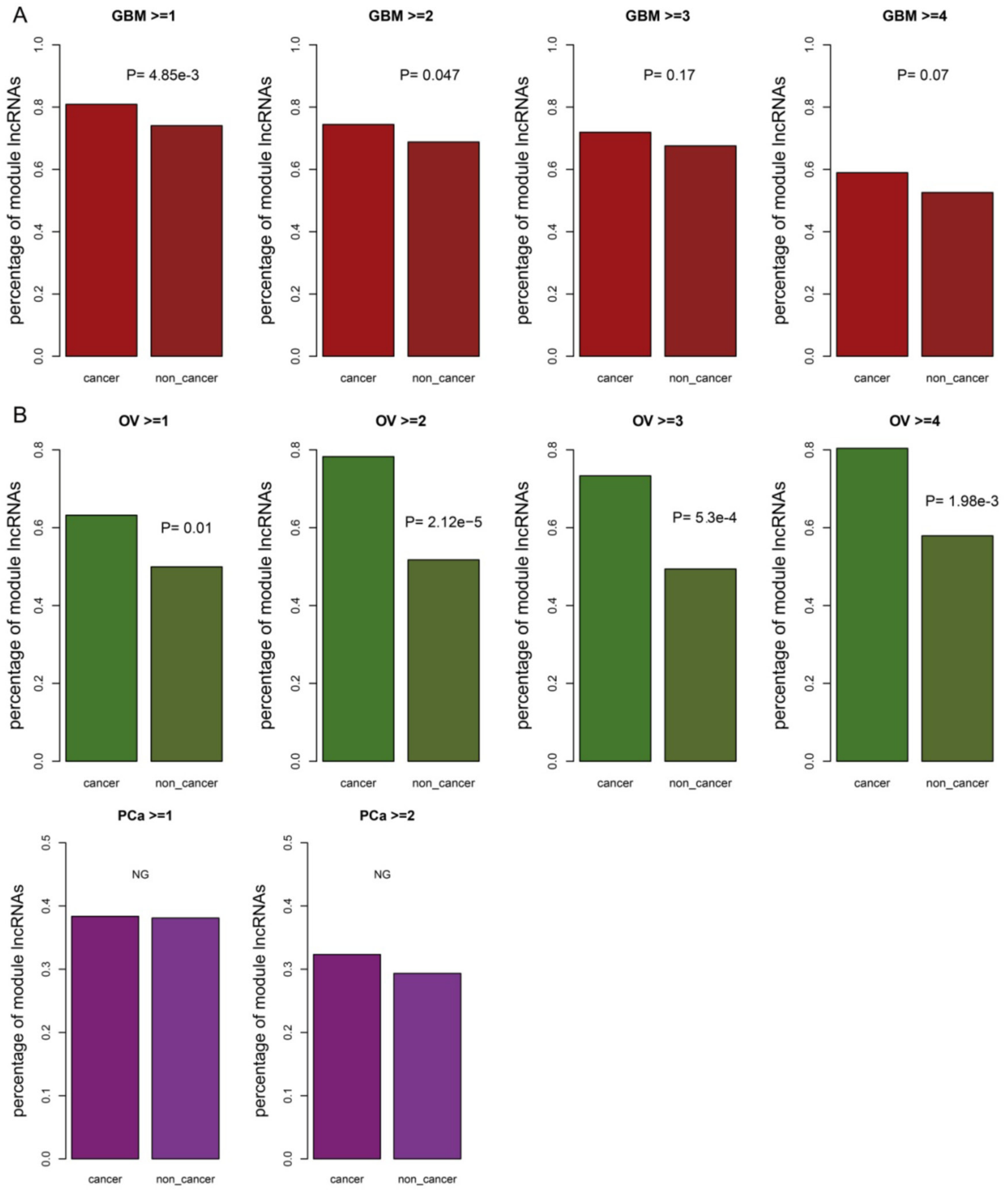

Supplementary Figure S7: The cancer associated lncRNAs are enriched in the module of weighted networks in GBM, OV and PCa. The subplots show the results of LFSNs at different threshold of the weights.

Supplementary Table S1: The lncRNA synergetic networks in three cancers

Supplementary Table S2: The results of multivariate survival analysis of lncRNA modules in GBM

**Module-1**	**HR(95% CI)**	***p*-values**	**Module-2**	**HR(95% CI)**	***p*-values**
**Cluster**			**Cluster**		
Cluster-1	1 (reference)		Cluster-1	1 (reference)	
Cluster-2	0.65 (0.46, 0.94)	**0.021**	Cluster-2	1.42 (1.11, 1.82)	**0.005**
**Age**	1.00 (1.00, 1.00)	8.57e-9	**Age**	1.00 (1.00, 1.00)	1.38e-8
**Gender**			**Gender**		
Female	1 (reference)		Female	1 (reference)	
Male	1.15 (0.93, 1.42)	0.21	Male	1.12 (0.90, 1.38)	0.31
**Subtypes**			**Subtypes**		
Classical	1 (reference)		Classical	1 (reference)	
Proneural	1.72 (1.24, 2.40)	0.001	Proneural	1.49 (1.11, 2.02)	0.009
Mesenchymal	1.05 (0.80, 1.38)	0.72	Mesenchymal	0.88 (0.65, 1.18)	0.39
Neural	1.13 (0.82, 1.55)	0.46	Neural	0.98 (0.71, 1.35)	0.88
G-CIMP	0.71 (0.42, 1.21)	0.21	G-CIMP	0.55 (0.34, 0.88)	0.01
**Module-3**	**HR(95% CI)**	***p*-values**	**Module-4**	**HR(95% CI)**	***p*-values**
**Cluster**			**Cluster**		
Cluster-1	1 (reference)		Cluster-1	1 (reference)	
Cluster-2	1.30 (1.03, 1.65)	**0.03**	Cluster-2	1.40 (0.99, 1.98)	**0.058**
**Age**	1.00 (1.00, 1.00)	7.12e-9	**Age**	1.00 (1.00, 1.00)	2.81e-8
**Gender**			**Gender**		
Female	1 (reference)		Female	1 (reference)	
Male	1.08 (0.87, 1.35)	0.49	Male	1.17 (0.94, 1.45)	0.16
**Subtypes**			**Subtypes**		
Classical	1 (reference)		Classical	1 (reference)	
Proneural	1.39 (1.03, 1.88)	0.03	Proneural	1.77 (1.22, 2.56)	0.002
Mesenchymal	0.94 (0.71, 1.26)	0.68	Mesenchymal	1.07 (0.81, 1.40)	0.64
Neural	1.09 (0.80, 1.50)	0.58	Neural	1.11 (0.81, 1.53)	0.51
G-CIMP	0.56 (0.35, 0.90)	0.02	G-CIMP	0.70 (0.40, 1.22)	0.21

Supplementary Table S3: The results of multivariate survival analysis of lncRNA modules in OV

Module-1	HR(95% CI)	*p*-values	Module-2	HR(95% CI)	*p*-values
**Cluster**			**Cluster**		
Cluster-1	1 (reference)		Cluster-1	1 (reference)	
Cluster-2	0.70 (0.55, 0.89)	**0.004**	Cluster-2	1.36 (1.06, 1.73)	**0.014**
**Age**	1.00 (1.00, 1.00)	9.81e-5	**Age**	1.00 (1.00, 1.00)	0.0003
**Grade**			**Grade**		
G1/G2	1 (reference)		G1/G2	1 (reference)	
G3/G4	1.23 (0.89, 1.68)	0.20	G3/G4	1.28 (0.93, 1.75)	0.13
**Stage**			**Stage**		
Stage I/II	1 (reference)		Stage I/II	1 (reference)	
Stage III	2.47 (1.26, 4.83)	0.008	Stage III	2.27 (1.15, 4.47)	0.02
Stage IV	3.47 (1.69, 7.11)	0.0007	Stage IV	3.23 (1.58, 6.70)	0.001

Supplementary Table S4: The prognosistic associated lncRNA modules in GBM and OV

Supplementary Table S5: The TF-gene/lncRNA regulations in GBM, OV and PCa

	TFs	lncRNAs	regulations	TFs	genes	regulations
**ChIPBase**	108	5,068	66,698	120	17,608	546,903
**GBM**	64	1,513	3,829	74	15,017	111,673
**OV**	64	998	2,147	74	13,854	74,696
**PCa**	59	1,960	4,869	74	15,107	106,160
